# Categorization and discrimination of human and non-human primate affective vocalizations: Investigation of frontal cortex activity through fNIRS

**DOI:** 10.1162/imag_a_00480

**Published:** 2025-02-20

**Authors:** Coralie Debracque, Leonardo Ceravolo, Zanna Clay, Didier Grandjean, Thibaud Gruber

**Affiliations:** Department of Psychology and Educational Sciences and Swiss Center for Affective Sciences, University of Geneva, Geneva, Switzerland; Department of Psychology, Durham University, Durham, United Kingdom

**Keywords:** recognition, affect, vocalization, primate, frontal, NIRS

## Abstract

Previous research has highlighted the involvement of frontal regions in human participants while they engaged in the explicit decoding, such as categorization (A*vs*B) and discrimination (A*vs*non-A), of affective signals. Given its adaptive value and deep evolutionary history, this human capacity to recognize the affective content in human calls is likely to extend to the vocalizations of other closely related species, such as non-human primates. However, few comparative studies have thus far investigated this process at both the behavioral and neural levels. Here, we aimed to study the role of frontal regions in human participants while they engaged in the explicit affective content decoding of primate calls using functional Near Infrared Spectroscopy (fNIRS). Specifically, we recorded frontal regions of participants while they categorized or discriminated positive and negatively valenced vocal signals produced by four different primates: humans, chimpanzee and bonobo (both great apes species), and rhesus macaques (a more distant species). We also analyzed whether behavioral responses correlated with recorded frontal activations. fNIRS data revealed more activations within the inferior frontal cortex*pars triangularis*(IFC_tri_), the frontopolar (FPC), and middle frontal cortices (MFC) during discrimination compared with categorization. Activity in these regions was modulated by both the species and the type of task, with greater activity during the discrimination of agonistic chimpanzee calls compared with categorization. Categorization was itself characterized by a decrease of frontal activity during the correct recognition of all chimpanzee calls, and of affiliative rhesus macaque and agonistic bonobo vocalizations. Our results also highlighted behavioral differences related to the type of task. Participants discriminated almost all affective cues of all four species vocalizations above chance level. In comparison, they correctly categorized the affective content of most human and great ape vocalizations above chance level, but not those of rhesus macaque calls, highlighting an effect of both phylogenetic relatedness and the type of task. Overall, these findings support the hypothesis of an evolutionary ancient affective recognition processing system situated in the frontal cortex, inherited from our last common ancestor with other great apes.

## 1 Introduction

While often associated with irrational choices, emotions play an essential role in guiding cognitive processes to enable adaptive responses to the environment (Brosch et al., 2013). Over the last three decades, psychologists (for a review, seeLerner et al., 2015) and neuroscientists (for a review, seePhelps et al., 2014) have investigated the impact of emotions on decision-making processes. Far from being limited to humans, there is also a deep evolutionary history to such affective mechanisms. By allowing animal species to evaluate others’ social motivations (Albuquerque et al., 2016) and react adaptively to the emotional valence of a situation (Mendl & Paul, 2020), affective recognition mechanisms are crucial for survival (Anderson & Adolphs, 2014;Filippi et al., 2017).

Every day, animals (humans included) receive emotional information conveyed by a range of modalities, including visual or auditory ones. Yet, visual cues are often compromised by distance or rich environments (Ghazanfar & Santos, 2004). As such, in the course of evolution, the vocal apparatus has become a privileged channel for the transmission and the recognition of emotions for a large number of species. For example, comparative research on mongooses (*Suricata suricatta*) and monkeys (*Cercopithecus diana; Cercopithecus nictitans martini*) has demonstrated the capacity of these species to modulate their alarm calls depending on the predator category, that is, aerial*vs*terrestrial (Arnold et al., 2008;Manser, 2001;Zuberbühler, 2000). In decoding the referential information conveyed by the alarm calls about the predator types, recipients can, therefore, react differently and then adopt the best strategy to maximize their chance of survival (Arnold et al., 2008;Fichtel & Kappeler, 2002).

From this evolutionary basis of vocal emotions, we can question the current existence of behavioral and brain mechanisms shared by modern humans (*Homo sapiens*) and other species, especially great apes, our closest relatives, for the recognition of affective vocalizations. In fact, as members of the*Hominidae*clade, which appeared between 13 and 18 million years ago (Perelman et al., 2011), humans share with the other living great apes (chimpanzees—*Pan troglodytes*, bonobos—*Pan Paniscus*, gorillas—*Gorilla*species, and orangutans—*Pongo*species) a long and common evolutionary history. If ancient emotional processing mechanisms inherited from our common ancestor are still at play, modern humans should in theory be able to correctly identify vocal emotions expressed by other great apes.

Yet, only a handful of behavioral and neuroimaging studies have investigated this question and results are currently inconsistent. In fact, if some findings confirm the crucial role of phylogenetic proximity to humans in the recognition of emotions in non-human primates (NHP) vocalizations, other results disagree as to whether modern humans can correctly identify affective cues in NHP calls. For instance, Kamiloğlu and colleagues demonstrated the ability of human participants to accurately identify most of the affective contexts in chimpanzee vocalizations (Kamiloğlu et al., 2020). However, participants seemed unable to do so for affective calls expressed by macaques (*Macaca mulatta*)—a more phylogenetically distant species to humans (Belin, Fecteau, et al., 2008;Fritz et al., 2018). These results tend to confirm the phylogenetic hypothesis, that is, that humans can only identify emotions expressed by other great apes. Moreover, functional magnetic resonance imaging (fMRI) data suggested that this recognition, regardless of the type of task, would rely on cortical activations in frontal cortex areas, especially in the inferior frontal cortex (IFC;Belin, Fecteau, et al., 2008;Ceravolo et al., 2023;Fritz et al., 2018). Frontal regions are in fact well known for their roles in decision-making and emotional processes in humans (Brück et al., 2011;Grandjean, 2020) as well as in other primate species (e.g. macaques;Barbas, 2000;Barbas et al., 2011;Binder et al., 2004;Davidson, 1992;Kambara et al., 2018;LeDoux, 2012).

On the contrary, other studies combining behavioral and electroencephalogram (EEG) aspects suggest a different picture. For example, human participants were not able to recognize affective cues in chimpanzee calls due to their poor familiarity with this species compared with dogs (*canis lupus familiaris*) or humans: the authors suggested a link between their behavioral results and the elicitation of posterior P3a and P3b, which are a marker of novelty processing at the brain level (Scheumann et al., 2014,^2017^). In contrast, Linnankoski and colleagues highlighted the ability of human adults and infants to classify most of the affective contents in macaque vocalizations (Linnankoski et al., 1994). Interestingly, recent findings using functional Near Infrared Spectroscopy (fNIRS) demonstrated that human affective recognition performance tends to be influenced by the primate species producing the vocalizations, that is, in terms of phylogenetic and acoustic proximity as well as by the type of recognition task, that is, categorization or discrimination, drawing a more complex picture to the origin of such mechanism (Debracque et al., 2023).

Therefore, it is still unclear whether modern humans are capable of recognizing affective cues in NHP vocalizations, especially those of great apes and if primate species, humans included, still share some affective mechanisms inherited from a common ancestor. In particular, disentangling previous results is necessary (Ackermann et al., 2014;Nieuwburg et al., 2021). The present paper attempts to fill this gap by combining a neuroscientific and behavioral approach to investigate human affective recognition processing in response to human and other primate vocalizations. Adult human participants performed categorization and discrimination tasks on the affective contents (agonistic*vs*affiliative) in human, great apes (chimpanzee, bonobo), and monkey (rhesus macaque) vocalizations, while their frontal brain activity was being recorded non-invasively using fNIRS. We explain these choices below.

First, this study distinguished categorization (unbiased choice, “A*vs*B”) and discrimination (biased choice, “A*vs*non-A”) mechanisms at play in frontal brain regions for the human recognition of affects in NHP vocalizations. In fact, previous data have shown that the categorization and the discrimination of affective cues in voices involve different behavioral and frontal cortex activity processes (Dricu et al., 2017). These distinct mechanisms related to the type of task could explain the difference of recognition rate for affective macaque calls between Linnankoski and colleagues who explicitly asked their participants to categorize, that is, classifying affective contexts (Linnankoski et al., 1994)*vs*the other studies involving the rating of valence on a visual analogue scale (Belin, Fecteau, et al., 2008;Fritz et al., 2018). Overall, more controlled investigations in this domain are thus needed (Gruber & Grandjean, 2017).

Second, based on the existing literature investigating the neural correlates of affective recognition in voice by human participants, we chose to focus this study on frontal cortex activity and especially on the IFC. In fact, while the processing of conspecific vocalizations in humans and non-human primate species strongly involved the temporal cortex (e.g.Barbas, 2000;Belin, 2006), studies on heterospecific recognition and identification of affect tend to demonstrate an important role of the IFC in such mechanisms (e.g.Belin, Fecteau, et al., 2008;Ceravolo et al., 2023;Fritz et al., 2018;Grandjean, 2020;Gruber et al., 2020). The*pars triangularis*of the IFC (IFC_tri_) appears particularly of interest since fMRI model-based and conjunction analyses have recently shown that the implication of the human IFC_tri_was anti-correlating with the fitted probability of accurate classification of primate affective vocalizations. Interestingly, the IFC_tri_was also found to be involved in categorization task while the discrimination of affective human voices involved the*pars opercularis*of IFC in adult humans (IFC_oper_;Dricu et al., 2017).

Third, to investigate whether the phylogenetic proximity plays a role in human vocal decoding of emotions expressed by primates, we included four primate species calls, including less studied bonobos. In fact, despite their affiliation to the great apes family and 98.7% of their DNA shared with humans (Prüfer et al., 2012), bonobos have singular evolutionary roots, undergoing a process of self-domestication (Hare et al., 2012), leading them to acoustically and behaviorally differ from chimpanzees (Debracque et al., 2023;Grawunder et al., 2018;Gruber & Clay, 2016). Moreover, only recognized as a separate species from chimpanzees in 1929 (Coolidge Jr., 1933), bonobos are still largely unknown to the general public. Crucially, involving this species allows disentangling various factors involved in recognition. Indeed, if only the phylogenetic proximity to humans explained the ability of participants to identify affective cues in other primate vocalizations, they should be capable to do so only for great apes (chimpanzee and bonobo) independently of the documented differences between these two species.

Finally, to disentangle the potential impact of emotional valence in recognition mechanisms, agonistic as well as affiliative vocalizations were included for all species. Indeed, it is well known in humans that negative screams, due to their evolutionary relevance for survival (Arnal et al., 2015), are recognized faster and better than joyful voices for instance (Schaerlaeken & Grandjean, 2018). Furthermore, neuroimaging studies using fMRI or fNIRS have demonstrated differences of activation for the processing of negative and positive voices in bilateral frontal regions such as IFC (Johnstone et al., 2006;Zhang et al., 2018). As such, adding positive and negative valence vocalizations to the current paradigm was crucial.

Overall, the aim of the present study was to investigate human participants’ ability to recognize affective contents in phylogenetically close or distant primate species through distinct perceptual decision-making processes using fNIRS to assess the role that frontal regions and in particular the IFC play in such mechanisms. Specifically, we were interested in testing the role that phylogenetic proximity as well as the type of task plays in modulating such processes. First, according to the*type of task*hypothesis, we predicted that the categorization task should involve a lower recognition rate and more activations in the IFC compared with other frontal regions than discrimination for which the highest level of correct answers should be found. Second, according to the*phylogenetic relatedness*hypothesis, we expected that the frontal regions and especially the IFC as well as the participants’ recognition rate would be modulated differently across human, great ape, and monkey vocalizations, with the following gradient: human > chimpanzee, bonobo > rhesus macaque calls. Finally, if the IFC is necessary to the recognition of affects in primate calls, its neural activity should be greater related to the participants’ performances than the other frontal regions which would be explained by the interaction between both the*phylogenetic relatedness*and*type of task*hypotheses.

## 2 Material & Methods

### 2.1 Participants

Thirty healthy adult volunteers (12 males; mean age 25.06 years, SD = 5.09, age range 20–36) took part in the experiment. While we did not run a power analysis because no previous study involving heterospecific vocalizations with such paradigm was available to predict effect sizes, we based our sample size on a previous study run in our research group (Gruber et al., 2020), which used a similar paradigm with human vocalizations only. This sample size was in line with current fNIRS studies on emotion at the time (Westgarth et al., 2021). The participants were undergraduate and postgraduate students from the University of Geneva. They reported normal hearing abilities and normal or corrected-to-normal vision. No participant presented a neurological or psychiatric history, or a hearing impairment, or had any prior training about the experimental task. All participants gave informed and written consent for their participation in accordance with the ethical and data security guidelines of the University of Geneva. The study was approved by the Ethics Cantonal Commission for Research of the Canton of Geneva, Switzerland (CCER).

### 2.2 Vocalization stimuli

Ninety-six vocalizations of four primate species (human, chimpanzee, bonobo, rhesus macaque) produced in agonistic and affiliative contexts were used as stimuli (for examples seeFig. 1). The human voices, obtained from the Montreal Affective Voices (Belin, Fillion-Bilodeau, et al., 2008), were denoted as expressing a happy, angry, or fearful affect (posed short emotional interjections using the vowel «ah») produced by five male and five female actors.

**Fig. 1.**
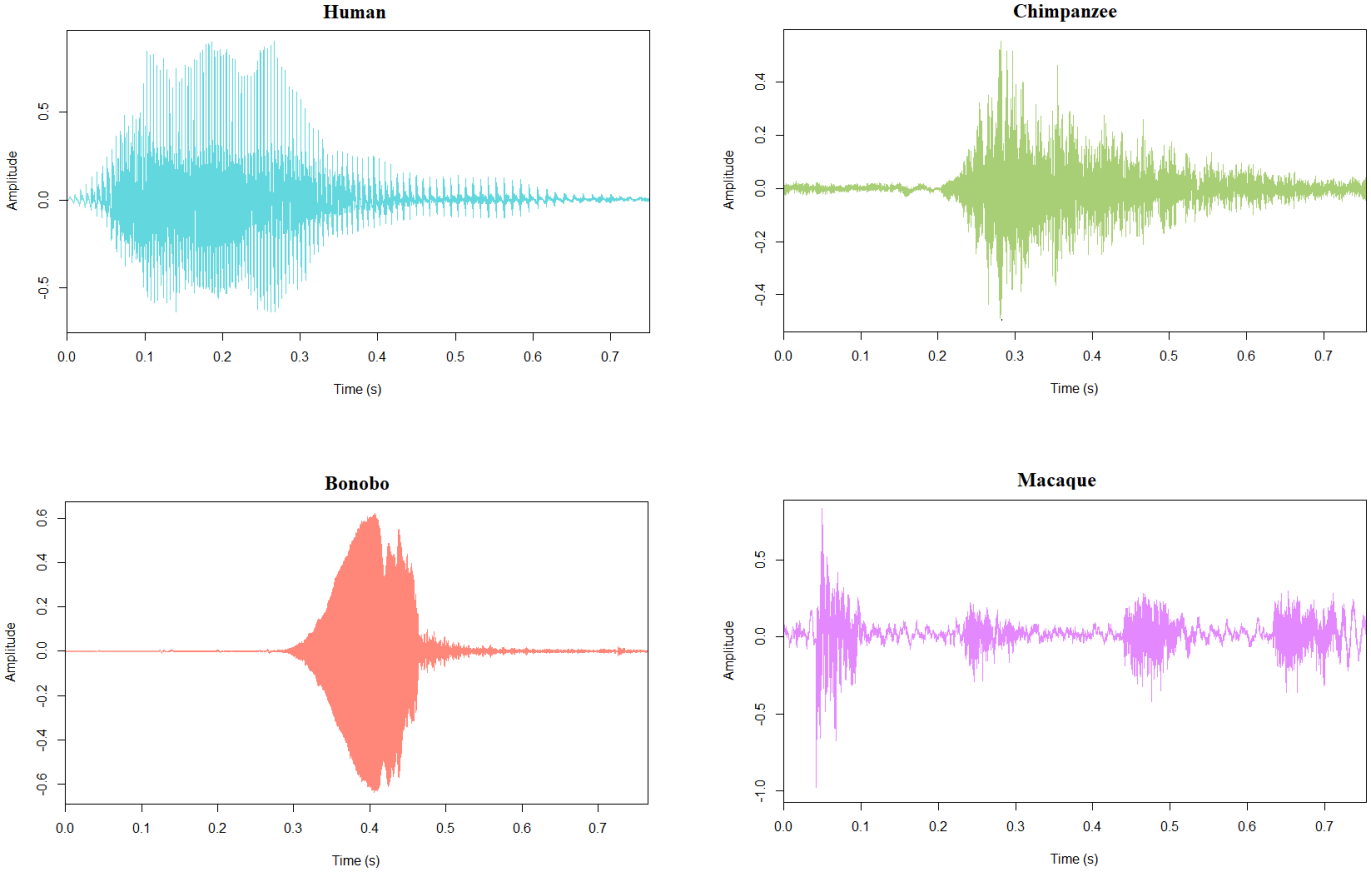
Representative waveforms of 750-ms-long angry/threatening vocalizations expressed by human (in blue), chimpanzee (in green), bonobo (in orange), and rhesus macaque (in pink) species. These graphical representations were extracted using the PhonTools package (Barreda, 2015) in Rstudio (Rstudio Team, 2020).

Vocalizations of corresponding affective categories were selected for chimpanzees, bonobos, and rhesus macaques under the form of affiliative calls (food grunts), threatening calls (aggressor in an agonistic context), and distress calls (victim in an agonistic context), which are commonly used in the literature to study primate vocalizations expressed in happy (positive), angry, and fearful (negative) contexts, respectively (Briefer, 2012;Kret et al., 2020). Systematic research has demonstrated that feeding and agonism reliably elicit multiple behavioral and physiological indicators of positive and negatively valenced emotion, respectively (Briefer, 2012,^2018^). In addition, research with the species under investigation (rhesus macaque and the Pan apes) has also further shown that these contexts reliably elicit acoustically distinct vocalizations that convey both affective and referential information to receivers about the nature of the event, including the social roles in a conflict (aggressor and victim) and the features of the food (Clay et al., 2016;Gouzoules et al., 1998;Slocombe & Zuberbühler, 2005). In a previous study, we also verified the acoustic properties of these stimuli underlying the affective vocalizations of these contexts in these species, and further showed that their affective context can be largely discriminated by naïve participants (Debracque et al., 2023). For each species, calls were selected by vocalization experts, with call selection involving acoustic verification through examination of spectral properties that conformed to parameters in existing repertoires. For each species, 24 stimuli were selected containing single calls or call sequences produced by 6 to 8 different individuals in their natural social environment.

All vocal stimuli were standardized to 750 ms using PRAAT (www.praat.org), but the maximum amplitude was not normalized in order to preserve the naturalness of the sounds (Ferdenzi et al., 2013). In fact, the amplitude distribution of mammalian calls strongly depends on both, the stimulus context (Lesica & Grothe, 2008), and the emotional state of the caller (Briefer, 2012). Normalizing the maximum amplitude of the vocal stimuli could, therefore, bias the recognition of emotions by human participants.

### 2.3 fNIRS acquisition

fNIRS is a non-invasive technique to study the brain hemodynamic (Boas et al., 2014) using the principle of tissue transillumination (Bright, 1831). In the present study, fNIRS data were acquired using the Octamon device (Artinis Medical Systems B.V., Elst, The Netherlands) at 10 Hz with 6 transmitters and 2 receivers (wavelengths of ±760 nm and ±850 nm) with inter-distance probes at 3.5 cm. The headband holding the 8 channels was placed identically for all participants according to the 10-20 electroencephalogram (EEG) system (Jasper, 1958;Okamoto et al., 2004) by using FPZ, F3, F4, F7, F8 as landmarks (seeFig. 2). These landmarks were taken by placing an EEG cap on the head of each participant beforehand. The probe locations into the Montreal Neurological Institute (MNI) space were estimated by using SPM12 software implemented in MatLab R2018b (www.fil.ion.ucl.ac.uk/spm/) and confirmed by the existing literature on EEG electrode positions (Koessler et al., 2009;Scrivener & Reader, 2022). Hence, the channels 1, 2, 7, and 8 were located on the*pars triangularis*of IFC (IFC_tri_, Broca’s area) and the channels 3, 4, 5, and 6 on the frontopolar (FPC) and middle frontal cortices (MFC). Note that other frontal regions could not be targeted because of the headband and the limited number of optodes available with the Octamon device.

**Fig. 2.**
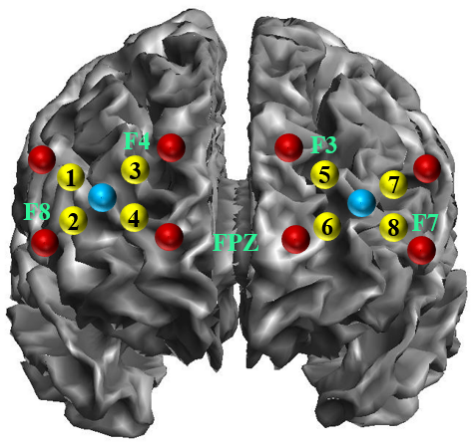
Probe locations into the MNI space by using SPM12 software implemented in MatLab R2018b (www.fil.ion.ucl.ac.uk/spm/). Red and blue dots indicate transmitters and receivers’ positions, respectively. Yellow dots indicate the channel numbers.

### 2.4 Experimental procedure

Seated comfortably in front of a computer, participants listened to the vocalizations played in stereo using Seinnheiser headphones at 70 dB SPL. Each of the 96 stimuli was repeated 9 times across 6 separate blocks leading to 864 trials following a randomization process. The overall experiment was structured in various layers (seeFig. 3). Testing blocks were task specific, with participants either performing a categorization task (A*vs*B) or a discrimination task (A*vs*non-A) in a single block, see below for more information. Participants completed three categorization blocks and three discrimination blocks, resulting in six blocks in total. Each block was made of 12 mini-blocks, each separated by a break of 10 s. These mini-blocks comprised one unique mini-block per species (human, chimpanzee, bonobo, and rhesus macaque), each mini-block repeated three times. Within each mini-block were 12 trials, containing 4 vocalizations from all 3 affective contexts (threat/anger; distress/fear; affiliative/happy) produced by a single species. The blocks, mini-blocks, and stimuli were pseudo-randomly assigned for each participant to avoid more than two consecutive blocks, mini-blocks, and stimuli from the same category.

**Fig. 3.**
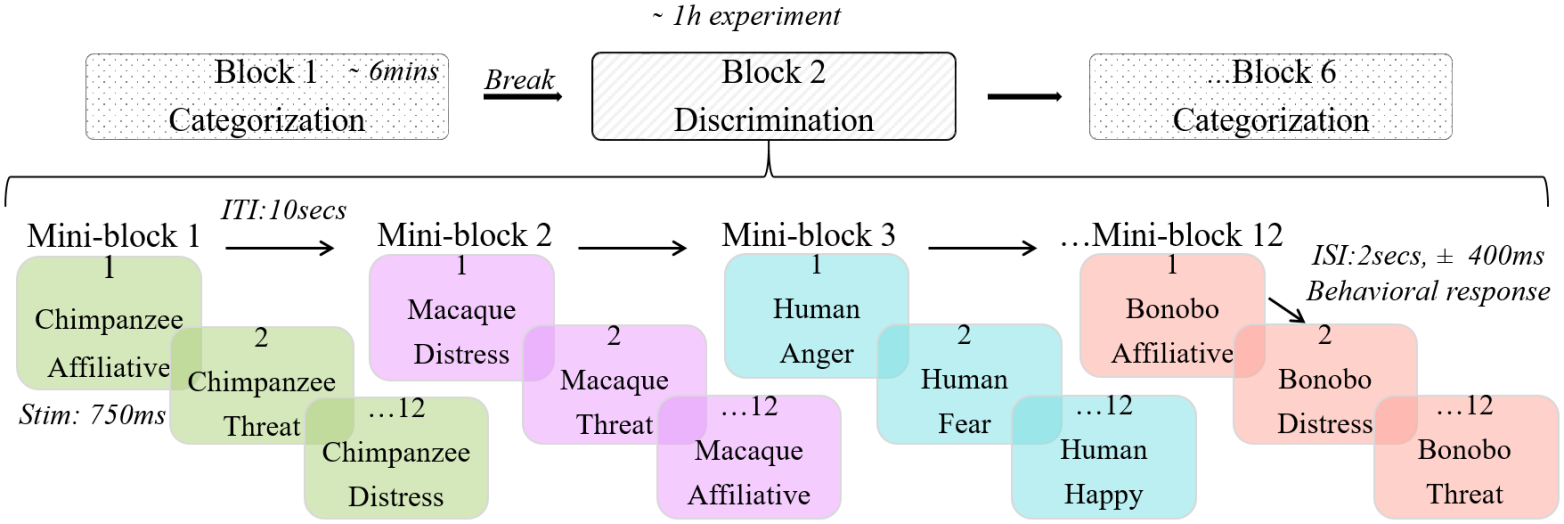
Structure of the experiment, with each of the 6 blocks made of 12 mini-blocks, which in turn comprised 12 individual trials.

At the beginning of each block, participants were instructed to identify the affective content of the vocalizations using a keyboard. For instance, the instructions for the categorization task could be “For Affiliative/Happy—press M; for Threatening/Anger—press Z; and for Distress/Fear—press space bar”. Similarly, the instructions for discrimination could be “For Affiliative/Happy—press Z and for other affect—press M”. The pressed keys were randomly assigned across blocks and participants. The participants had to press the key during the 2-s intervals (randomization of intervals time variation, i.e., jittering of 400 ms) between each stimulus. If the participant did not respond during this interval, the next stimulus followed automatically.

### 2.5 Statistical analysis

#### 2.5.1 Behavioral data

Raw behavioral data were analyzed using Generalized Linear Mixed Model (GLMM). The following three factors and their interactions were included: Stimuli species (human, chimpanzee, bonobo, and rhesus macaque), Tasks (categorization—CAT and discrimination—DIS), and Affect Type (threat/anger, distress/fear, affiliative/happy). Participant ID and block order were included as random effects. We first tested the full model against a null model containing only intercept and random effects. Second, we tested whether each fixed factors and then all three factors explain a significant part of variance. Third, the interaction models were run. For all GLMMs, the more complex model was systematically tested against the less complex one (dropping one fixed factor or in the case of the interactions tested against the models including the fixed factors and their intercepts) with an Akaike information criterion (AIC) check for which smaller values indicate better models. The models were fitted by Restricted Maximum Likelihood (REML) in Rstudio (Rstudio Team, 2020) with the “bobyqa” function (optimization by quadratic approximation with a set maximum of 1,000,000 iterations) and the “logit” link for a standard logistic distribution or errors and a binomial error distribution (correct answer—1 or not—0) of the package Lme4 (Bates et al., 2015). To test our hypotheses regarding the*phylogenetic relatedness*and the*type of task*on participant performance, we compared the differences between Species and Affect Type within the categorization and discrimination tasks. These contrasts were corrected with Bonferroni correction (P_corrected_= .05/24 = .002). Similarly, participant reaction time (correct answers only) was analyzed using a GLMM with a Gaussian distribution with the same contrasts and analyses. The present paper focusing on the investigation of recognition mechanisms, not attentional processes, results for reaction times are reported inSupplementary Material.

#### 2.5.2 fNIRS data

In line with previous power analyses in fMRI (Desmond & Glover, 2002) and research using fNIRS to assess emotional processing in frontal areas (for a review, seeBendall et al., 2016), data from N = 20 participants were analyzed in this study. Ten of the original 30 participants were excluded due to poor signal quality (N = 5; confounding signals) or missing fNIRS data (N = 5; recording problem due to technical issues). The fNIRS signal processing pipeline was as follows:

1. In order to limit confounding signals in our data, we performed a first level analysis on all channels with preprocessing steps using a General Linear Model (GLM) approach on the SPM-fNIRS toolbox (Tak et al., 2016,^2008^;https://www.nitrc.org/projects/spm_fnirs/):
  ○ Hemoglobin concentration changes were calculated with the modified Beer–Lambert law (Delpy et al., 1988) using a differential pathlength factors (DPF) correction for each participant.
  ○ Motion artifacts were reduced using the movement artifact reduction algorithm (MARA—Scholkmann et al., 2010) based on moving standard deviation and spline interpolation.
  ○ Systemic and physiological confounds such as cardiac modulation, respiration, and vasomotion usually found in extra-cerebral blood flow were reduced using a high-pass filter based on a discrete cosine transform set with a cutoff frequency of 1/64 Hz (Friston et al., 2000) and a precoloring method using a low-pass filter based on the hemodynamic response function (HRF—Friston et al., 2000). The use of both filtering enables a better signal–noise ratio than conventional methods (Patashov et al., 2023). Note that the use of short channels was not feasible because of the fixed headband of the Octamon device.
2. In order to include the maximum peak amplitude of the HRF observed across participants, O_2_Hb concentration changes were averaged between 4 and 12 s post-stimulus onset on each trial using our own Matlab scripts (Version 2028b;The MathWorks Inc., 2009). As for fMRI imaging, this method of analysis taking into account the slow hemodynamic time course of brain activity is in line with the literature using auditory stimuli in fNIRS (e.g.Lloyd-Fox et al., 2014).

Following the same procedure as for behavioral data, the second level analysis was performed on Rstudio using GLMM fitted by REML with the factors: Stimuli Species (human, chimpanzee, bonobo, rhesus macaque), Task (categorization versus discrimination), Affect type (threat/anger, distress/fear; affiliative/happy), as well as their interactions as fixed factors. Participant ID and block orders were included as random factors for the right and left IFC_tri_and FTC/MFC. Note that because our study was especially interested by the role of IFC compared with other frontal regions, data for FTC and MFC were not analyzed separately.

#### 2.5.3 Interaction between participant performance and brain oxyhemoglobin (O _2_ Hb) changes

To test whether the frontal activations facilitated recognition accuracy, we used fNIRS data as continuous predictors in GLMM analysis performed on Rstudio for accuracy. To perform this statistical interaction, we only used accuracy from the 20 participants included in fNIRS analyses. The GLMM fitted by REML included Stimuli Species (human, chimpanzee, bonobo, and rhesus macaque), Task (discrimination and categorization), Affect Type (threat/anger, distress/fear, affiliative/happy,) as fixed factors, fNIRS data from the right and left IFC_tri_and FTC/MFC as continuous predictors, and participant ID as a random factor. To assess the variance explained by the phylogeny as well within the frontal activation, we tested all slopes with the following contrast: human*vs*[great apes (chimpanzee and bonobo)]*vs*rhesus macaque. We then assessed how the affective contents modulated IFC_tri_and FTC/MFC activity across species vocalizations during the categorization or discrimination tasks. For this purpose, we investigated whether the participants’ accuracy and the related fNIRS data were positively, negatively, or not correlated for each species and ROIs within the Affects and Tasks factors using odds ratio.

## 3 Results

### 3.1 Accuracy

We investigated how the perceptual decision-making complexity influenced human participants’ ability to recognize affective contents in phylogenetically close or distant primate species (seeFig. 4). A GLMM analysis on mean recognition rate revealed that the full model including main effects and the interaction between Stimuli species, Task and Affect type explained significantly more variance compared with the null model (χ^2^(23) = 3355.9, p < .001). Statistical values of GLMM models are reported inTable 1.

**Fig. 4.**
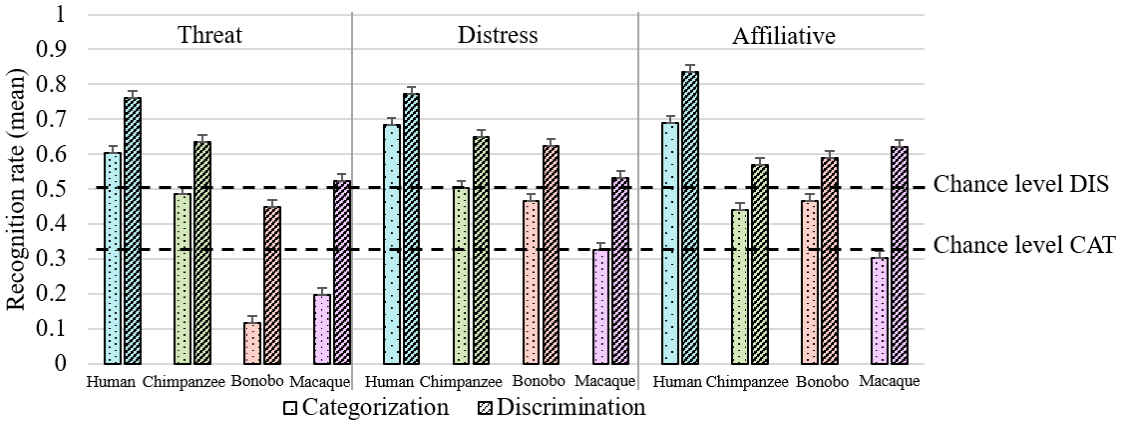
Mean and SE of human recognition of human (in blue), chimpanzee (in green), bonobo (in orange), and rhesus macaque (in pink) affective vocalizations for categorization (CAT) and discrimination (DIS) tasks and the different kinds of affective states. All contrasts were significant within each condition after Bonferroni correction with P_corrected_= .05/24 = .002, excluding the following contrasts: chimpanzee*vs*rhesus macaque and bonobo*vs*rhesus macaque for affiliative cues and bonobo*vs*rhesus macaque for threatening contents in discrimination task (seeSupplementary Material Table 2).

**Table 1.** Table summarizing the statistical values for the GLMM of mean recognition rate including main effects and the interaction.

Summary of the model for accuracy	Df	Chi-squared	p-value
Stimuli species	3	734.36	<.001
Task	1	1626.3	<.001
Affect type	2	129.34	<.001
Stimuli species: task: affect type	6	82.165	<.001

According to one-sample t-test analyses, participants performed significantly above chance (>50% in discrimination; >33% in categorization) for the recognition of most of the affective cues in great ape vocalizations (excluding bonobo threat calls—seeTable 2for test statistics andSupplementary Material Table 1). However, they were unable to do so for rhesus macaque threat calls in the discrimination task and for all rhesus macaque affective vocalizations in the categorization task.

**Table 2.** Summary of the t-test statistics against chance level for N = 20 participants.

	Categorization			Discrimination		
	Threat/anger	Distress/fear	Affiliative/happy	Threat/anger	Distress/fear	Affiliative/happy
*Bon*	* t _(19)_* *=* *-5.96* *p < .001*	***t*** **_(19)_** **=** **3.68** **p** * < * **.01**	***t*** **_(19)_** **=** **3.33** **p** * < * **.01**	* t _(19)_* *=* *-1.12* *p* *=* *.28*	***t*** **_(19)_** **=** **4.49** **p** * < * **.001**	***t*** **_(19)_** **=** **3.08** **p** * < * **.01**
**Chimp**	***t*** **_(19)_** **=** **3.63** **p** * < * **.01**	***t*** **_(19)_** **=** **4.35** **p** * < * **.001**	***t*** **_(19)_** **=** **3.27** **p** * < * **.01**	***t*** **_(19)_** **=** **5.00** **p** * < * **.001**	***t*** **_(19)_** **=** **3.55** **p** * < * **.01**	***t*** **_(19)_** **=** **3.12** **p** * < * **.01**
**Hum**	***t*** **_(19)_** **=** **8.62** **p** * < * **.001**	***t*** **_(19)_** **=** **9.99** **p** * < * **.001**	***t*** **_(19)_** **=** **32.94** **p** * < * **.001**	***t*** **_(19)_** **=** **10.96** **p** * < * **.001**	***t*** **_(19)_** **=** **11.60** **p** * < * **.001**	***t*** **_(19)_** **=** **23.65** **p** * < * **.001**
**Mac**	* t _(19)_* *=* *-2.30* *p < .05*	* t _(19)_* *=* *0.69* *p* *=* *.50*	* t t _(19)_* *=* *0.61* *p =.55*	* t _(19)_* *=* *1.50* *p =.15*	***t*** **_(19)_** **=** **2.40** **p** * < * **.05**	***t*** **_(19)_** **=** **4.44** **p** * < * **.001**

Recognition performance above chance (>33% categorization and >50% discrimination with p* < *.05) is given in bold. Recognition performance significantly under (p* < *.05) or equal (p > .05) to chance level is given in italic.

Abbreviations: bonobo (Bon), chimpanzee (Chimp), human (Hum), and rhesus macaque (Mac).

Following this, as predicted by the*type of task*hypothesis, contrasts after Bonferroni correction (P_corrected_=.002) in the three-way interaction showed that participants were better at discriminating than categorizing affective vocalizations expressed by all primate species with humans: (χ^2^(1) = 145.72, p < .001), chimpanzees: (χ^2^(1) = 138.86, p < .001), bonobos: (χ^2^(1) = 327.74, p < .001), and rhesus macaques: (χ^2^(1) = 546.73, p < .001). Regarding the*phylogenetic relatedness*hypothesis, contrasts also revealed that human participants categorized and discriminated better (i) human voices compared with NHP calls for threat/anger: χ^2^(1) = 558.13; distress/distress: χ^2^(1) = 292.84 and affiliative/happy: χ^2^(1) = 445.9, p < .001; (ii) great apes [chimpanzee and bonobo] vocalizations compared with rhesus macaque ones for threat: χ^2^(1) = 13.66; distress: χ^2^(1) = 105.21 and affiliative: χ^2^(1) = 18.56, p < .001; and (iii) threatening chimpanzee compared with threatening bonobo calls (χ^2^(1) = 374.57, p < .001). Note that no significant difference was found between these two great ape species for distress (χ^2^(1) = 4.59, p = .03) and affiliative vocalizations (χ^2^(1) = 2.39, p = .1).

### 3.2 fNIRS data

A GLMM analysis on fNIRS data (all channels) revealed that the full model including main effects and the interaction between Stimuli species, Task, and Affect type explained significantly more variance compared with the null model (χ^2^(23) = 121.1, p < .001). Moreover, as expected by the type of*task*hypothesis, statistics showed a significant main effect of Task in the right IFC_tri_(χ^2^(1) = 14.27, p < .001), left IFC_tri_(χ^2^(1) = 3.89, p < .05), right FTC/MFC (χ^2^(1) = 107.32, p* < *.001), and left FTC/MFC (χ^2^(1) = 90.83, p < .001) revealing more O_2_Hb concentration changes for the discrimination compared with the categorization task for all ROIs (seeFig. 5). Overall, in both tasks, the bilateral IFC_tri_was positively activated compared with the bilateral FTC/MFC that were deactivated. Note that none of the interactions with the factors Affect type and Stimuli species reached significance.

**Fig. 5.**
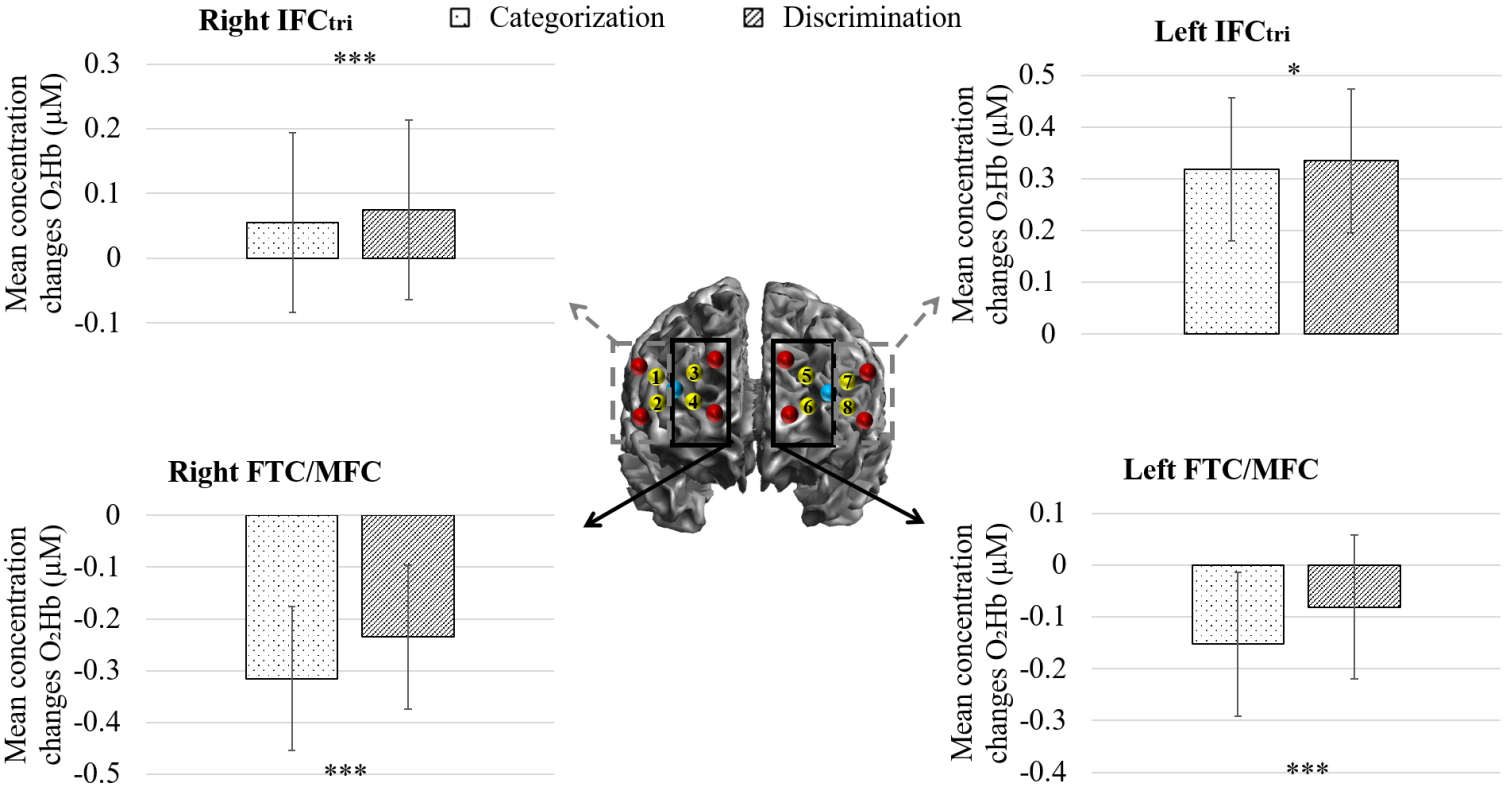
Mean and SE of concentration changes of O_2_Hb (µM) in right and left FTC/MFC and IFC_tri_during the categorization and the discrimination tasks by human participants of primate affective vocalizations. N = 20. ***p < .001, *p < .05.

### 3.3 Interaction between participant performance and brain O _2_ Hb changes as measured by fNIRS

In order to test whether activity in the IFC compared with the two other frontal regions facilitates or not the human recognition of affects in primate vocalizations, we first run GLMM analyses to reveal the factors that were potentially in play in such mechanisms. We found that all factors (Task, Stimuli species, and Affect type) with the fNIRS data of the right and left IFC_tri_and FTC/MFC as continuous predictors contributed to a significant three-way interaction (χ^2^(24) = 202,28 p < .001). In addition, the full model including main effects and the interaction between Stimuli species, Task, Affect type, and fNIRS data as continuous predictor have shown to explain significantly more variance compared with the null model (χ^2^(30) = 150.89, p < .001).

Second, odds ratio measuring the relationship between the recognition performance and the frontal activity (seeTable 3) showed that participants better discriminated agonistic (threat and distress) chimpanzee calls when the concentration changes of O_2_Hb increased in IFC_tri_and FTC/MFC. At the opposite, during the categorization task, the correct identification of all types of chimpanzee calls as well as affiliative rhesus macaque and agonistic bonobo vocalizations was associated with a decrease of activity in frontal regions. Moreover, we tested whether phylogenetic proximity facilitated the recognition of Affect. We found for both frontal regions that contrasts between humans*vs*[great apes (chimpanzees and bonobos)]*vs*rhesus macaques within each Affect and Task were significant at p < .001 (seeSupplementary Material Table 4). Note that because we found similar patterns of performance between the frontal regions, for more clarity, we only describe the results for IFC_tri_here (seeFig. 6). Results for FTC/MFC are reported inSupplementary Material Figure 3.

**Table 3.** Summary of the odds ratio and p-values testing the statistical significance and the direction of logistic regression slopes from the three-way interaction.

	Categorization			Discrimination		
	Threat	Distress	Affiliative	Threat	Distress	Affiliative
Bonobo	**0.84** *	**0.88** *	1.06	0.99	1.1	1.06
Chimpanzee	**0.78** *	**0.69** **	**0.86** *	***1.28*** *	***1.44*** **	0.93
Human	1.02	1.13	1.11	0.98	0.89	1.02
Rhesus macaque	1.07	0.94	**0.85** *	0.93	0.9	1.05

The odds ratio quantifies the strength of the association between two factors. If the slope is significant and odds ratio < 1, factors are negatively correlated (written in bold); if the slope is significant and odds ratio > 1, factors are positively correlated (written in bold italic). ** p < .01, * p < .05.

**Fig. 6.**
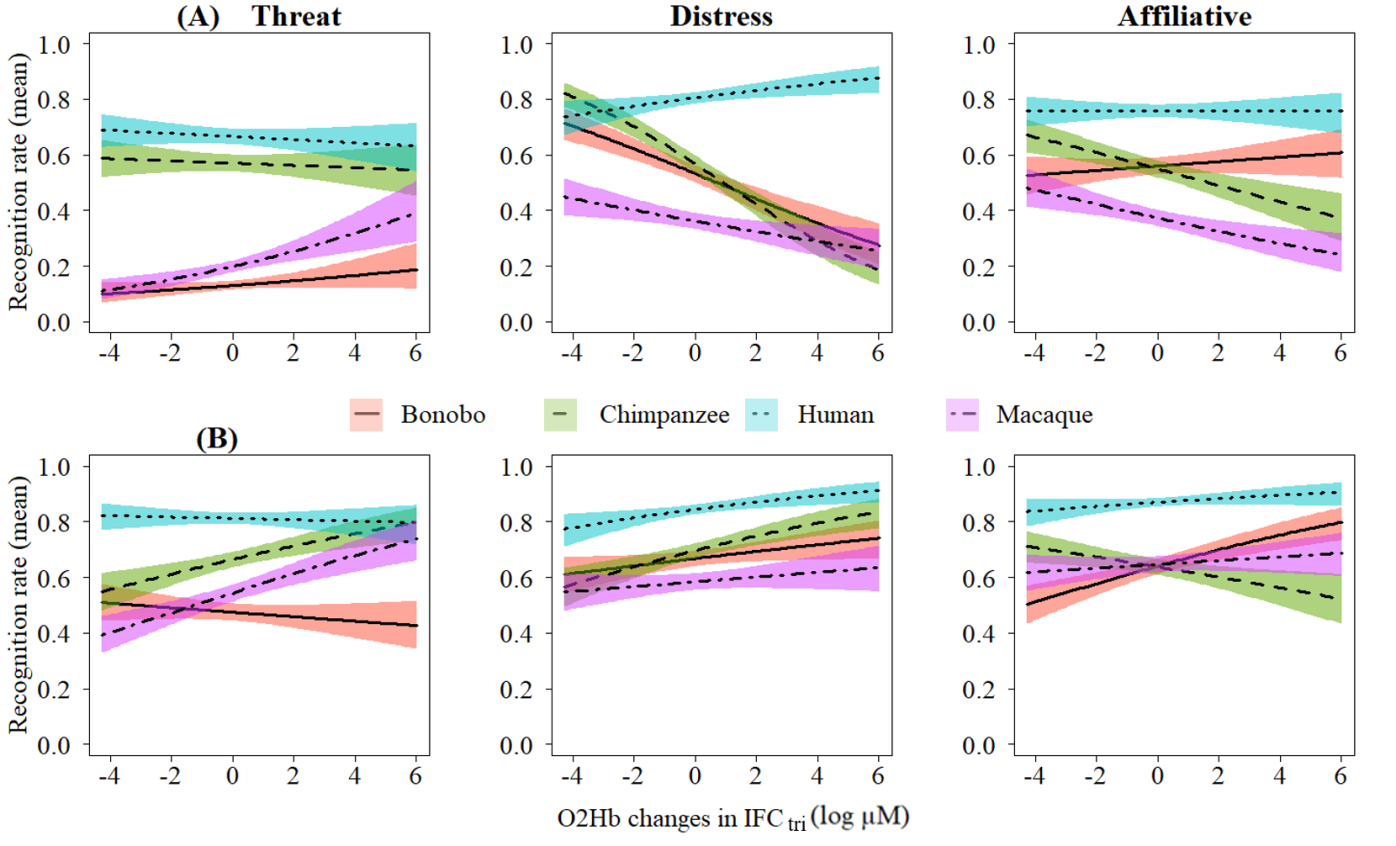
Interaction between participants’ accuracy and O_2_Hb concentration changes in IFC_tri_within each affect and species for (A) categorization and (B) discrimination. Confidence interval at 0.95. Figures were made on Rstudio using the package Visreg (Breheny & Burchett, 2017).

## 4 Discussion

The present study investigated how human participants are capable of recognizing affective contents in phylogenetically close or distant primate species through distinct perceptual decision-making mechanisms using a combined behavioral and neuroscientific approach. First, by using a two-task design (categorization and discrimination), we demonstrated that the IFC_tri_was differently activated in both tasks compared with the two other investigated frontal regions (namely FTC and MFC). In addition, we found that these frontal cortex areas were more involved in the discrimination task than in the categorization task, with participants overall better at discriminating affective calls from all species than categorizing them. Second, considering the phylogenetic relatedness of primates including vocalizations expressed by great ape and monkey species, we showed that participants were better at recognizing human emotional voices, then great ape affective calls (from chimpanzees and bonobos) and then, rhesus macaque vocalizations for which the lowest accuracy was found. Interestingly, fNIRS data also revealed a modulation of activity in IFC_tri_and FTC/MFC depending on the phylogenetic proximity to humans. Finally, we also found that the*type of task*and*phylogenetic relatedness*mechanisms did interact with each other to affect the recognition of affective cues in primate vocalizations at the brain and behavioral level.

The existing literature on human voices has shown that the categorization and the discrimination of vocal affective cues indeed involve different distinct recognition mechanisms with a greater level of correct answers for discrimination (biased choice) than for categorization (unbiased choice;Debracque et al., 2023;Dricu et al., 2017;Gruber et al., 2020). Based on this, we expected in the present study a role of the*type of task*on the brain and behavioral mechanisms at play in the recognition of primate affective calls by human participants with a lower recognition rate for categorization comparing with discrimination. This justified the use of*type of task*as a fixed factor in all our models. As predicted, we did find in our behavioral results that participants were better at discriminating than categorizing affective cues expressed by all primate species. Furthermore, the mechanisms involved in the discrimination task seem to enable human participants to even correctly identify affective vocalizations expressed by rhesus macaques, a phylogenetically distant species to humans, while they were unable to do so in categorization.

On the contrary, fNIRS data also demonstrated a stronger involvement of the IFC_tri_and the two other frontal regions for discrimination compared with categorization, which was not expected based on the literature. Indeed, we previously demonstrated using different imaging technique and/or paradigm, that the IFC, and especially the bilateral IFC_tri_compared with the other frontal regions were strongly implicated in the categorization of human and non-human primate affective voices (Ceravolo et al., 2023;Gruber et al., 2020). However, these results for IFC_tri_are coherent with the ones found by Dricu and collaborators, showing that the IFC_tri_is particularly involved in the discrimination of human affective voices while the IFC_oper_is more involved in the categorization task. Overall, we might hypothesize that a modulation in frontal cortex areas would enable participants to perform better during the discrimination of primate vocalizations. This hypothesis is supported by our results in the interaction between participant performance and brain O_2_Hb changes as measured by fNIRS in which we found that the more their IFC_tri_and FTC/MFC were activated, the more human participants accurately discriminated agonistic (threat and distress) vocalizations expressed by chimpanzees.

Interestingly, fNIRS data also revealed a general positive activation for IFC_tri_compared with a decrease of activity for FTC/MFC in response to affective vocalizations. The decrease of O_2_Hb concentration changes in FTC/MFC could be linked to the changes in regional cerebral blood flow. Indeed, Matsukawa and collaborators showed using fNIRS that during the passive viewing of emotional videos (horror or comedy movies featuring humans), the activity in PFC regions such as FTC and dorsolateral prefrontal cortex (DLPFC) decreased in correlation to the reduction of facial skin blood flow (Matsukawa et al., 2018). These authors suggested that PFC activity might elicit an autonomic reaction with a vasoconstriction or a vasodilatation of cutaneous vessels. In the same line, George and collaborators demonstrated a stronger decrease of activity in right PFC, especially in right MFC and DLPFC regions during the viewing of pleasant pictures, also relying on a reduction of the frontal blood flow (George et al., 1995). A possibility is thus to extend the results of these visual studies to a decrease of activity in FTC/MFC regions during affective auditory processing.

Overall, these results highlight the distinct frontal cortex and behavioral mechanisms at play in humans for the discrimination and categorization of affective primate calls.

Was human recognition influenced by the phylogenetic relatedness of the species that expressed the vocalizations? Our results suggest that the phylogenetic proximity to humans influenced participant behavioral responses and its interaction with frontal activations. In fact, as expected by the*phylogenetic relatedness*hypothesis, our behavioral data have shown that participants better recognized (categorization and discrimination tasks including) human emotional voices and then great ape affective calls (expressed by chimpanzees and bonobos—our closest relatives) compared with rhesus macaque affective vocalizations for which the lowest recognition rate was found. Moreover, human participants were mostly unable to identify correctly, that is, above chance level, affective cues in rhesus macaque calls. These results are supported by the slopes analysis investigated interaction between participant performances and brain O_2_Hb changes. In fact, data revealed that frontal activations underlying the correct emotional recognition of human voices were closer to the activations linked to the identification of great ape vocalizations compared with rhesus macaque calls. These last data are coherent with recent fMRI findings highlighting the crucial role of IFC and OFC in the human recognition of great ape vocalizations (Ceravolo et al., 2023). Overall, these results may highlight the phylogenetic gap of 25–33 million between rhesus monkeys and the*Hominidae*branch (Perelman et al., 2011). Interestingly, despite that acoustic properties of the vocalizations are intrinsically linked to the phylogeny of the species, our behavioral data are not influenced by the vocal amplitude or loudness for instance of the stimuli (seeDebracque et al., 2023, for more details). While we acknowledge that we did not normalize the maximum amplitude of the stimuli, potentially leading to an influence on our results, we believe that a normalization could potentially alter the ecological acoustic relevance of the stimuli, and preferred to keep them as recorded. Nevertheless, vocal amplitude or loudness is only one of many acoustic parameters that may influence our results, and the present study was not intended nor designed to investigate such, often subtle, variation. We encourage future research to study in detail the links between these mechanisms and acoustic variables.

Restricting ourselves to great apes only, strikingly, our behavioral analyses also demonstrated that participants could discriminate threatening calls expressed by chimpanzees but not the ones from bonobos. In line with our previous study in humans showing the role of both phylogenetic and acoustic similarity in the recognition of affects in non-human primate vocalizations (Debracque et al., 2023), we hypothesize that specific acoustic factors in bonobo calls triggered this effect. Indeed, bonobo calls have a higher fundamental frequency resulting from a shorter vocal length in comparison with chimpanzees (Grawunder et al., 2018). In this species, signaling physical strength using low frequencies (e.g.Briefer, 2012;Morton, 1982) is not a sexually selected trait (Grawunder et al., 2018). This is reflected in their general behavior and neuroanatomical traits (Staes et al., 2018), with bonobos being quite different from closely related chimpanzees and overall less aggression prone: they are occasional hunters, do not have strict territories and have a developed socio-sexuality, reducing the number of aggressive conflicts (Gruber & Clay, 2016). Furthermore, threatening vocalizations are structurally different from those produced in distressful or affiliative contexts in mammals (Morton, 1977) with threat having for instance a lower average frequency (pitch) comparing with distress (Scherer, 2003;Sobin & Alpert, 1999). In the present study, the high frequencies conveyed by threatening bonobo calls seem to prevent human participants from correctly identifying this emotional cue. For example, Kelly and colleagues have already demonstrated that the very high pitched of bonobo vocalizations compared with the lower pitch of chimpanzees biases human participants in their recognition of emotional intensity in agonistic bonobo vocalizations (Kelly et al., 2017).

Overall, these results point out the crucial role of phylogenetic proximity in the categorization and discrimination of affective primate calls by humans. However, other factors such as the acoustic properties of the vocalizations also seem to be involved in such mechanisms.

Finally, due to the existing literature on categorization and discrimination tasks described earlier (Debracque et al., 2023;Dricu et al., 2017;Gruber et al., 2020) and on the ability of modern humans to correctly identify chimpanzee affective calls but not the ones expressed by macaques (Belin, Fecteau, et al., 2008;Fritz et al., 2018;Kamiloğlu et al., 2020), we expected more activity in the IFC_tri_compared with the other two frontal regions underlying the accurate recognition of affective vocalizations by adult humans. We hypothesized a modulation of frontal cortex activity and participants’ performances depending on the interaction between both the*type of task*and*phylogenetic relatedness*to humans. Our results showed that while the correct discrimination of agonistic chimpanzee calls was underlined by an increase of O_2_Hb concentration changes in the investigated frontal regions, the accurate categorization of all chimpanzee vocalizations, affiliative rhesus macaque, and agonistic bonobo calls by participants was related to a decrease of O_2_Hb in the IFC_tri_as well as in the FTC and MFC. Therefore, to the exception of affiliative rhesus macaque calls that were recognized by participants as well as affiliative and chimpanzee and bonobo calls, interaction between participants’ performance and frontal activations was only found for great apes highlighting an influence of phylogenetic proximity on primate affective calls recognition. Moreover, distinct mechanisms between the categorization and discrimination tasks seem also involved in such process. In fact, the significant decrease of activity in the IFC_tri_, FTC, and in the MFC elicited by a correct categorization of bonobo and chimpanzee vocalizations might be related to an inhibition process enabling participants to reduce a high level of stress elicited by these unusual calls, that is, automatic regulation. Frontal regions are indeed the most sensitive brain areas to stress exposure (Arnsten, 2009). Possible inhibition processes would rely on a decrease of activations in frontal regions for the simple choice between A*vs*non-A; while in categorization (A*vs*B), similar inhibition mechanisms would require an enhancement of activity in IFC_tri_and FTC/MFC. In contrast, we propose that the general absence of an interaction between frontal activations and explicit detection of affective content of human voices might be explained by the fact that voices in our modern human societies are everywhere (Belin, 2006), and thus, the correct recognition of vocal affects may not strongly involve frontal regions due to the human expertise in conspecific voice processing (Belin, 2006). Indeed, it is particularly well known in fMRI that experts compared with naïve human participants show a poorer activity in the task-related regions underlying their skills and providing evidence of neural efficiency (Bernardi et al., 2013;Jeon & Friederici, 2017).

To conclude, and related to the*type of task*hypothesis, we first demonstrated that the frontal cortex regions were strongly involved in the discrimination task compared with the categorization one. From a behavioral perspective, participants were overall better at discriminating affective calls than categorizing. Second, considering the*phylogenetic relatedness*hypothesis, we showed that human participants were better at recognizing human emotional voices, then great ape affective calls and then rhesus macaque vocalizations for which the lower accuracy was found. Interestingly, fNIRS data also revealed a modulation of activity in the frontal regions depending on the phylogenetic proximity to humans. Our findings demonstrate the interplay between cerebral and behavioral processes during the recognition by humans of affective cues in primate vocalizations. The*type of task*and*phylogenetic relatedness*seem essential markers to consider for further studies on affective primate recognition, as our results highlight the interaction between the two at both the behavioral and brain levels. Overall, we demonstrated the difference of mechanisms between the categorization and discrimination of primate affective vocalizations. In particular, we showed various activations in the frontal regions with a distinct involvement of the inferior frontal cortex (IFC_tri_) compared with the FTC (frontopolar cortex) and MFC (middle frontal cortex) and their connection to the ability of humans to correctly identify affective cues in great apes’ vocalizations. Furthermore, our results highlighted the importance of the phylogenetic proximity and also suggest a role of acoustic properties in affective recognition processes. Finally, to our knowledge, this study is the first to (i) distinguish categorization and discrimination processes in a study with a comparative perspective and (ii) assess the link between primate affective recognition and frontal activations in an fNIRS paradigm. The present study has, however, a few limitations. For instance, we focused only on the frontal cortex. Investigation of the fronto-temporal network would, therefore, be interesting for future studies as both cortices are strongly involved in such mechanisms. Following this, we explored IFC_tri_activity but not the other subparts of IFC such as*pars orbitalis*or*pars opercularis*(IFC_oper_). In particular, the IFC_oper_seems to be of interest with its involvement in categorization task. Eventually, due to the fixed probes on the fNIRS device headband, the use of short channels was not feasible. While we assessed confounding factors in our fNIRS data with relevant and validated processing steps, we are aware that the use of short channels is currently the best method to remove such artifacts. Despite these limitations, as well as the ones discussed above, we believe that our new findings contribute to a better understanding of the evolutionary origins of emotional processing and decision making in humans, as well as advocate for the inclusion of a broader array of auditory stimuli.

## Supplementary Material

Supplementary Material

## Data Availability

Data and codes used in the present study are available at a public and non-profit repository:https://yareta.unige.ch/,https://doi.org/10.26037/yareta:tuy5mbocbnf65p5hle44pqtvce.
